# Multi-Sensor Calibration of Low-Cost Magnetic, Angular Rate and Gravity Systems

**DOI:** 10.3390/s151025919

**Published:** 2015-10-13

**Authors:** Markus Lüken, Berno J.E. Misgeld, Daniel Rüschen, Steffen Leonhardt

**Affiliations:** Philips Chair for Medical Information Technology, RWTH Aachen University, Pauwelsstrasse 20, Aachen 52074, Germany; E-Mails: misgeld@hia.rwth-aachen.de (B.J.E.M.); rueschen@hia.rwth-aachen.de (D.R.); leonhardt@hia.rwth-aachen.de (S.L.)

**Keywords:** MEMS, calibration, magnetic, angular rate and gravity sensors, BSN

## Abstract

We present a new calibration procedure for low-cost nine degrees-of-freedom (9DOF) magnetic, angular rate and gravity (MARG) sensor systems, which relies on a calibration cube, a reference table and a body sensor network (BSN). The 9DOF MARG sensor is part of our recently-developed “Integrated Posture and Activity Network by Medit Aachen” (IPANEMA) BSN. The advantage of this new approach is the use of the calibration cube, which allows for easy integration of two sensor nodes of the IPANEMA BSN. One 9DOF MARG sensor node is thereby used for calibration; the second 9DOF MARG sensor node is used for reference measurements. A novel algorithm uses these measurements to further improve the performance of the calibration procedure by processing arbitrarily-executed motions. In addition, the calibration routine can be used in an alignment procedure to minimize errors in the orientation between the 9DOF MARG sensor system and a motion capture inertial reference system. A two-stage experimental study is conducted to underline the performance of our calibration procedure. In both stages of the proposed calibration procedure, the BSN data, as well as reference tracking data are recorded. In the first stage, the mean values of all sensor outputs are determined as the absolute measurement offset to minimize integration errors in the derived movement model of the corresponding body segment. The second stage deals with the dynamic characteristics of the measurement system where the dynamic deviation of the sensor output compared to a reference system is corrected. In practical validation experiments, this procedure showed promising results with a maximum RMS error of ${\text{3.89}}^{{}^{\circ}}$.

## 1. Introduction

Low-cost inertial (accelerometers and gyroscopes) and magnetic sensors have been used in various applications, ranging from consumer electronics to medicine [1]. These sensors are based on micro-electro-mechanical systems (MEMS) and are either available as 3D (alternatively 2D + 1D) integrated components or 9D integrated devices, offering on-board sensor fusion algorithms [2]. A device that provides information about the orientation, such as roll, pitch and yaw, is called an “attitude and heading reference system” (AHRS) in aerospace applications, yet other abbreviations exist, such as “magnetic, angular rate and gravity” (MARG) system. In biomedical or biomechanical applications, the size of the MARG sensor allows for easy attachment to the body or to a body segment. This opens new possibilities, like the independent processing of segmental orientation and short-time position that can be used in conjunction with a physically-constrained dynamic rigid body model. The result is a broad area of biomedical applications, which includes, for example, human locomotion [3,4], fall detection [5] and rehabilitation [6]. To obtain the orientation of the sensor node with respect to an inertial reference frame, vector observations from gravity and magnetic field measurements can be used and fused with gyroscopic measurements in, for example, an extended Kalman filter (EKF). Several recent studies, for instance [7,8,9], have been conducted on the solution of the data fusion problem. The performance of data fusion solutions, however, depends strongly on the quality of the sensor readings. Moreover, when such a data fusion solution is compared to a reference measurement of a secondary system (for example, an infrared motion capturing system), a successful alignment of the two inertial reference frames of the MARG sensor system and the reference system has to be guaranteed. Thus, in addition to an appropriate calibration of the MARG sensors, an employed calibration device or method should be able to minimize errors in the misalignment of reference frames. A calibration procedure should improve the quality of an orientation estimation algorithm, on the one hand, and increase the validity of reference measurements, on the other hand.

Various procedures for the calibration of inertial and magnetic sensors have been proposed in the literature. Ferraris *et al.* [10] presented a calibration procedure for the simultaneous calibration of 3D acceleration and gyroscope sensors only; the procedure is based on a set of simple rotations with a case that contains the sensors employing a reference surface. After manual rotation, a system of linear equations is solved to obtain sensor bias, scale factors and the orthogonalization matrix. In contrast to that, a procedure for stand-alone calibration of a 3D magnetometer that uses a calibration cube and a reference surface, similar to [10], was introduced by Cai *et al.* [11]. The calibration of inertial and magnetic sensors was presented in [12]. Here, a 9DOF MARG unit is placed into a case, and a calibration procedure is proposed for the accelerometer/magnetometer and the gyroscope. The gyroscope procedure is based on [10], where in case of the accelerometer/magnetometer procedure, the sensor is rotated about the roll axis and the yaw axis in two consecutive steps. In contrast to that, Bonnet *et al.* [13] introduced a procedure for the simultaneous calibration of inertial and magnetic sensors. Besides a mounting frame calibration procedure with simple in-plane movements, the sensor frame procedure is reformulated by an ellipsoid-fitting problem. Kok *et al.* [14] derived an easy-to-use calibration algorithm for MARG sensors. The approach relies on the use of probabilistic models and the solution of a maximum likelihood problem to compensate for static magnetic distortions created by the sensor platform, magnetometer sensor errors and alignment mismatch between magnetometer and inertial sensor axes. The authors extend the method in [15] by using a grey-box system identification to further simplify their previous approach. Grivon *et al.* [16] proposed an eigen-analysis approach for 3D-scanning applications based on an ellipsoid-fitting algorithm to estimate both sensor gains and the discrete cosine matrix (DCM), which aligns the ellipsoid to the chosen reference plane. Zhang and Yang [17] present a micro-magnetometer and inertial sensor calibration procedure that consists of a two-step approach to determine the combined bias and transformation matrix separately.

In contrast to the mentioned approaches, we present a calibration procedure that computes bias, scaling factors and orthogonalization matrix simultaneously. The procedure does not rely on pre-defined rotations; instead, only arbitrary movements are needed. Furthermore, a mounting frame and a reference platform are used to address the problem of aligning the inertial system of the MARG sensor with an inertial reference system. Finally, the three types of sensors are calibrated at once to increase the accuracy of the calibration procedure, leading to the solution of a multisensor calibration problem.

The paper is organized as follows. In Section 2, the calibration problem is formulated, and in Section 3, the calibration setup, as well as the calibration algorithm are presented. Simulation and experimental results of the procedure are detailed in Section 4. Finally, the paper ends with a discussion of the results in Section 5 and a conclusion in Section 6.

## 2. Problem Formulation

In many applications, like, for example, rehabilitation robotics or gait analysis, motion tracking of body segments is an important and critical task. In most cases, the technology of choice is optical tracking systems or high quality orientation measurement devices, which both are accompanied by high financial expenditure. In contrast to that, we intend to use a low-cost BSN-integrated IMU to estimate human body segment orientations. This information would be useful for many applications in rehabilitation robotics, as, for example, in joint-angle estimation in exoskeleton systems, in the analysis and identification of pathological gait patterns or in monitoring of the elderly, which are highly in danger of falling due to gait instabilities. The integration of MARG sensors into the “Integrated Posture and Activity Network by Medit Aachen” (IPANEMA) BSN structure also gives an advantage because of the already existing communication infrastructure. With the availability of low-cost MEMS sensor techniques, new approaches of data processing and calibration algorithms may accomplish approximately the same accuracy at much lower costs. This can only be achieved by sophisticated calibration and fusion algorithms that compensate the limitations of low-cost hardware. On the one hand, the sensors themselves have lower quality, resulting in higher bias, cross-axis misalignment or increased noise components; on the other hand, hardware implementation is limited in precision due to a lack of integration possibilities or misalignment caused by the soldering processes. To combine angular rate, acceleration and magnetic field sensors into one orientation estimation system, efficient algorithms need to be developed, which take into account all of these problems. The major aims of this contribution are:

- Starting from a previously-implemented quaternion-based orientation estimation algorithm [3], one task of this contribution is to derive an appropriate multi-sensor calibration procedure combining all three types of sensor characteristics utilizing a high quality inertial measurement unit (IMU) as the reference system.
- Since the reference orientation is available during the calibration process, this approach should not just focus on reference sensor outputs, such as acceleration, angular velocity and magnetic field strength, but also take into account the actual orientation to achieve higher accuracy.
- The determination of frame transformations is another problematic task due to the misalignments of the reference and body frame of the sensor itself. The fact that we use a mechanical fixing calibration aid simplifies this problem, but does not reduce its importance for calibration purposes.
- As the motion tracking should be performed for various individual body segment movements, the calibration algorithm should deliver optimal results for both static and highly dynamic motions. Therefore, the derived calibration procedure should be based on arbitrary movements, such that the expected motion dynamics can already be taken into account within this process.

**Figure 1:**
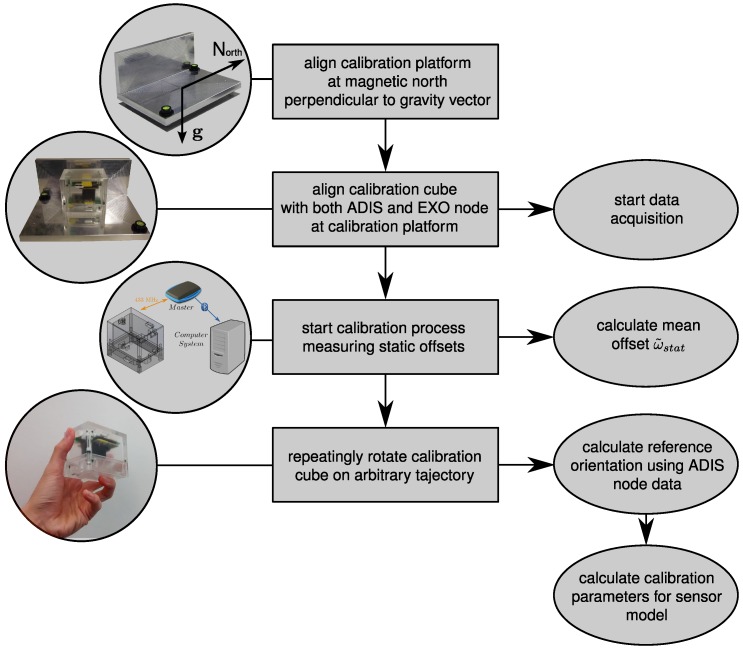
Overview of the calibration procedure with users actions depicted in rectangular boxes and algorithm parts in ellipses.

## 3. Methods and Materials

In order to utilize a recently-developed 9DOF sensor node for the purpose of body segment orientation estimation, the aim of this work was to derive a suitable calibration algorithm, since non-calibrated sensor systems may cause problematic deviations in orientation with respect to a reference system. This new approach for calibrating the developed low-cost MARG sensor system consists of basically two different procedures using the IPANEMA body sensor network (BSN) and our recently-constructed calibration cube. Figure 1 gives an overview of the overall calibration procedure, where the actions to be performed by the user are depicted in rectangular boxes on the left and the corresponding algorithm parts are shown on the right. In this section, we present the IPANEMA BSN, used for experimental validation, the sensor model and the calibration setup. Furthermore, our developed algorithm is discussed.

### 3.1. The IPANEMA Body Sensor Network

**Figure 2:**
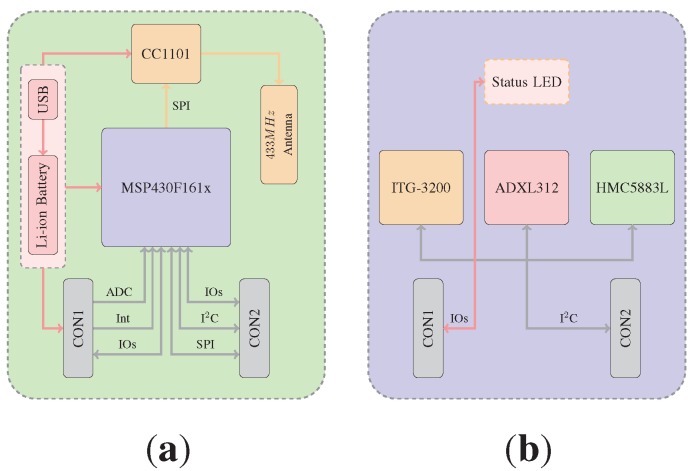
Integrated Posture and Activity Network by Medit Aachen (IPANEMA) design and components. (**a**) IPANEMA main board design; (**b**) IPANEMA 9DOF node design.

The IPANEMA BSN [18] was developed as a modular structure BSN that communicates between a master and multiple slave nodes by using the 433-MHz industrial, scientific and medical (ISM) band (CC1101, Texas Instruments Inc, Dallas, TX, USA). Allowing a maximum data rate of 250 kbps (which is sufficient for the typical activity and physiological parameter measurements), the advantage of the ISM band realized communication is the reduced susceptibility to the electromagnetic shadowing effects of the human body. An overview of the modular board structure is given in Figure 2. The interfaces CON1 and CON2 can be used to connect a slave node sensor modality or the master node communication board to the data collecting system. The IPANEMA BSN employs time division multiple access (TDMA) over a star-shaped network architecture. IPANEMA hardware and software design, for example the hardware abstraction layers (HAL) and the medium access control (MAC), guarantees a modular integration and the communication of various extension boards, carrying different sensing modalities. IPANEMA v2.5 is equipped with power management (LTC3558, Linear Technology, Milpitas, CA, USA), two extension ports (CLP160-02-X-D, Samtec, New Albany, IN, USA) and a microcontroller (MSP430F1611, Texas Instruments Inc., Dallas, TX, USA). Additionally, it provides an extension port (Microstac12, Erni Electronics GmbH, Adelberg, Germany) for custom functional units, including sensors and actuators.

Two extension boards were designed for IPANEMA BSN-based orientation estimation. The first extension board (EXO board) is equipped with triaxial inertial and magnetic sensors, as shown in Figure 2b. The inter-integrated circuit (I${}^{2}$C) bus with the microcontroller as the master and all of the sensors as slaves is used for on-board communication. The gyroscope (ITG-3200, InvenSense Inc., San José, CA, USA) and the acceleration sensor (ADXL312, Analog Devices Inc., Norwood, MA, USA) are based on MEMS technology; the magnetic field sensor (HMC5883L, Honeywell International Inc., Morristown, NJ, USA) uses magneto-resistive elements to measure the magnetic field strength. The second sensor node (ADIS16400 board) uses an integrated 9DOF inertial/magnetic sensor (ADIS16400, Analog Devices, Norwood, MA, USA). In Table 1, important parameters of the integrated sensors are listed for comparison. MEMS inertial and magneto-resistive sensing elements are small and provide low-cost and low-energy measurements of the gravitational acceleration, angular velocity and magnetic field strength. However, especially low-cost sensors still lack bias stability and have a comparatively high spectral noise. Therefore, in addition to sophisticated data fusion methods, a calibration procedure is necessary before a 9DOF MARG sensor system can be used.

**Table 1:** Specifications of both the reference and proposed BSN-integrated magnetic, angular rate and gravity (MARG) sensor system in comparison.

	ADIS16400			IPANEMA EXOBoard		
	Sensitivity	Range		Type	Range	Sensitivity
Gyroscope	0.05${}^{{}^{\circ}}$/s/LSB	±300${}^{{}^{\circ}}$/s		ITG-3200	0.07${}^{{}^{\circ}}$/s/LSB	±2000${}^{{}^{\circ}}$/s
Accelerometer	3.33 mg/LSB	±18 g		ADXL312	23.3 mg/LSB	±12 g
Magnetometer	0.5 mGauss/LSB	±3.5 Gauss		HMC5883L	0.73 mGauss/LSB	±0.88 Gauss
						

### 3.2. Sensor Modeling

The orientation of a rigid body in space is described by the transformation from an arbitrary body-fixed coordinate system, the so-called body frame *B*, to a static reference system, the navigation frame *N*, whose axes are fixed to the Earth’s geographic coordinate system. Based on physical characteristics, a common way to describe the reference system is the local geodetic frame, whose x-axis points to magnetic north, and the z-axis is in parallel down to the gravitational acceleration vector. Thus, the y-axis completes the right-handed reference system pointing to the east. Transformation of a vector $u\in R^{3}$ between two different coordinate systems is performed by a rotation matrix or the so-called direction cosine matrix (DCM) $C_{i}^{j}\in R^{3\times 3}$, where *i* and *j* depict the current and target system, such that:

(1) $$u^{n}=C_{b}^{n}\;u^{b}$$

The DCM in $R^{3}$ is defined as an orthonormal matrix, which obeys:

(2) $$\det\left(C\right)=1,\;C^{-1}=C^{T}$$

Note that the DCM can either be used to transform vectors between two systems or to characterize the body orientation relating to the chosen reference system. To achieve stability and increase computational efficiency in navigation tasks, utilization of the orientation quaternion description is an appropriate way, because of its non-singularity and simple mathematical manageability in comparison to other descriptions, like the Euler rotation, Euler angles (roll, pitch and yaw angle) or the rotation matrix given above. For the set of all quaternions H in the four-dimensional vector space, any single quaternion q∈H can be written as:

(3) $$q=\left[\begin{array}{c} q_{1}\\ q_{2}\\ q_{3}\\ q_{4} \end{array}\right]=\left[\begin{array}{c} q_{1}\\ \overset{˘}{q} \end{array}\right]$$

where $q_{1}$ depicts the scalar and $\overset{˘}{q}$ the vector part. As quaternions form a division algebra, their norm and conjugate are defined as:

(4) $$\begin{array}{cc} ∥q∥ & =\sqrt{q_{1}^{2}+q_{2}^{2}+q_{3}^{2}+q_{4}^{2}} \end{array}$$

(5) $$\begin{array}{cc} q^{*} & =\left[\begin{array}{c} q_{1}\\ -\overset{˘}{q} \end{array}\right] \end{array}$$

For the purpose of the orientation description, H is subdivided into the subset of unit quaternions $H_{1}$, which fulfil ∥q∥=1 and, thus, represent valid orientations. Using normed quaternions, the rotation of any vector $u\in R^{3}$ is simply performed by:

(6) $$\left[\begin{array}{c} 0\\ u^{n} \end{array}\right]=q⊗\left[\begin{array}{c} 0\\ u^{b} \end{array}\right]⊗q^{*}$$

where ⊗ denotes the non-commutative quaternion multiplication operator and $q\in H_{1}$ expresses the transformation from the *B* to *N* frame (body to navigation frame, respectively). The relation between the quaternion time derivative $\dot{q}$ and the navigation system-related angular velocity vector $\omega^{n}$ is described by the well-known identity:

(7) $$\left[\begin{array}{c} 0\\ \omega^{n} \end{array}\right]=2\dot{q}⊗q^{*}\;\Leftrightarrow \;\dot{q}=\frac{1}{2}\left[\begin{array}{c} 0\\ \omega^{n} \end{array}\right]⊗q$$

The body-referenced rotational velocity $\omega^{b}$ can be expressed by making use of the inverse rotation given in Equation (6):

(8) $$\left[\begin{array}{c} 0\\ \omega^{b} \end{array}\right]=q^{*}⊗\left[\begin{array}{c} 0\\ \omega^{n} \end{array}\right]⊗q$$

With Equation (7), it follows:

(9) $$\left[\begin{array}{c} 0\\ \omega^{b} \end{array}\right]=2q^{*}⊗\dot{q}⊗\underset{=1}{\underset{︸}{q^{*}⊗\;q\;}}=2q^{*}⊗\dot{q}$$

which finally leads to the sensor referenced angular velocity differential equation:

(10) $$\dot{q}=\frac{1}{2}q⊗\left[\begin{array}{c} 0\\ \omega^{b} \end{array}\right]$$

Since multiplication is non-commutative in quaternion algebra, it should be noted that Equation (10) does not equal Equation (7) in general.

**Figure 3:**
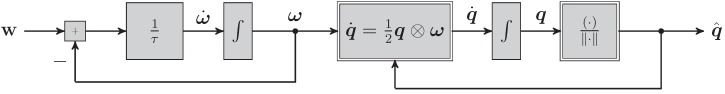
Model of human movement modified according to [19].

For the purpose of orientation estimation, the human movement is modeled by a non-linear state space system depicted in Figure 3, as given in [19]. The resulting non-linear state equations for the assumed human gait model are given as follows:

(11) $$\begin{array}{cc} {\dot{x}}^{\prime } & =\frac{1}{\tau }\left(w-x^{\prime }\right)\\ {\dot{x}}^{\prime \prime } & =\frac{1}{2}\;x^{\prime \prime }⊗\left[\begin{array}{c} 0\\ x^{\prime } \end{array}\right]\; \end{array}$$

with $x^{T}=\left[{x^{\prime }}^{T},{x^{\prime \prime }}^{T}\right]$ and $x^{\prime }=\omega$, $x^{\prime \prime }=q$. The additive input component $w\in R^{3}$ is assumed to be white Gaussian noise modeling the stochastic human movement.

**Figure 4:**
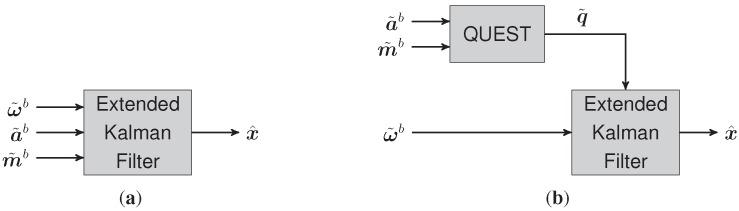
Block diagram of the extended Kalman filter design (**a**) in conventional MARG orientation estimation and (**b**) with quaternion estimator (QUEST) preprocessing.

In this approach, the acceleration vector $\tilde{a}$, as well as the magnetic field strength vector $\tilde{m}$, both obtained by the MARG sensor system, are preprocessed by the quaternion estimator (QUEST) algorithm [20] in order to avoid time-intensive calculation of the functional matrix as otherwise demanded by the stand-alone extended Kalman filter (EKF) approach. In contrast to that in this case the estimated quaternion vector $\tilde{q}$ is treated as an actual measurable quantity, as shown in Figure 4, resulting in the following linear measurement model for the EKF:

(12) z=x+v

where $v\in R^{7}$ is assumed to be zero-mean white Gaussian measurement-related noise. For a detailed description of QUEST, please refer to [20]. Finally, in order to model sensor inaccuracies, the measurement values are described by the following sensor model:

(13) $$\begin{array}{c} \begin{array}{cc} \tilde{\omega } & =K_{\omega }C_{n}^{b}\left(q\right)\omega^{n}+b_{\omega }+v_{\omega }\\ \tilde{a} & =K_{a}\left[C_{n}^{b}\left(q\right)\left(g^{n}+a^{n}\right)\right]+b_{a}+v_{a}\\ \tilde{m} & =K_{m}C_{n}^{b}\left(q\right)h^{n}+b_{m}+v_{m}\; \end{array} \end{array}$$

with matrices $K_{\omega }$, $K_{a}$, $K_{m}\in R^{3\times 3}$ containing scale factors, cross-axis sensitivity, soft-iron distortions (for magnetic sensors) and misalignment. The vectors $b_{\omega }$, $b_{a}$, $b_{m}\in R^{3}$ and $v_{\omega }$ denote bias and hard-iron offset vectors, respectively, and $v_{a}$, $v_{m}\in R^{3}$ uncorrelated zero-mean white Gaussian noise processes. Since quaternions are a convertible depiction of rotation matrices, the DCM can easily be calculated from:

(14) $$C_{}^{}\left(q\right)=\left(q_{1}^{2}-{\overset{˘}{q}}^{T}\overset{˘}{q}\right)I_{3}+2\overset{˘}{q}{\overset{˘}{q}}^{T}-2q_{1}{\left[\overset{˘}{q}\right]}_{\times }\;$$

with $I_{3}\in R^{3\times 3}$ identity matrix and:

(15) $${\left[\overset{˘}{q}\right]}_{\times }=\left[\begin{array}{ccc} 0 & -q_{4} & q_{3}\\ q_{4} & 0 & -q_{2}\\ -q_{3} & q_{2} & 0 \end{array}\right]$$

the skew-symmetric matrix of a vector product.

**Figure 5:**
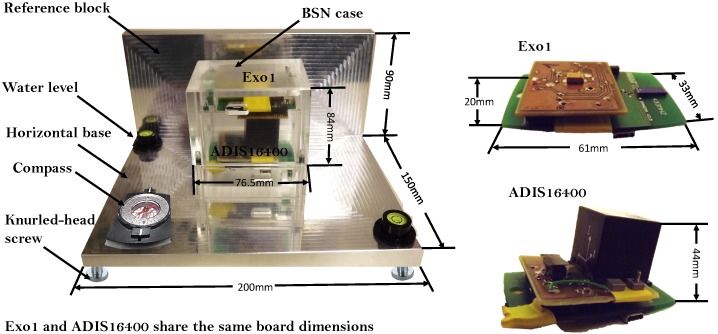
Overview of the components for sensor calibration.

### 3.3. Calibration System Design

A calibration system was developed for multi-sensor calibration of the 9DOF MARG sensors. The system was designed for improving existing calibration platforms [10] and consists of a reference platform (reference block and base) and a BSN case, shown in Figure 5. The horizontal base and block reference parts are made of precision-milled aluminum cast plate G.AL${}^{®}$ C250 (Gleich Group, Kaltenkirchen, Germany); the horizontal base and block reference part are perpendicularly aligned. To align the platform and thus the calibration cube with respect to the g-vector, it is furthermore fitted with knurled-head screws, positioned at the four corners. As indicators of an adequate adjustment, two auto-adhesive water levels (diameter 25 mm, height 10 mm, accuracy ±0.0286${}^{{}^{\circ}}$) are mounted on the horizontal base. A compass can be used to align the platform with respect to a certain heading, and thus, during the calibration procedure, the calibration cube can be aligned with the north-east-down system.

**Figure 6:**
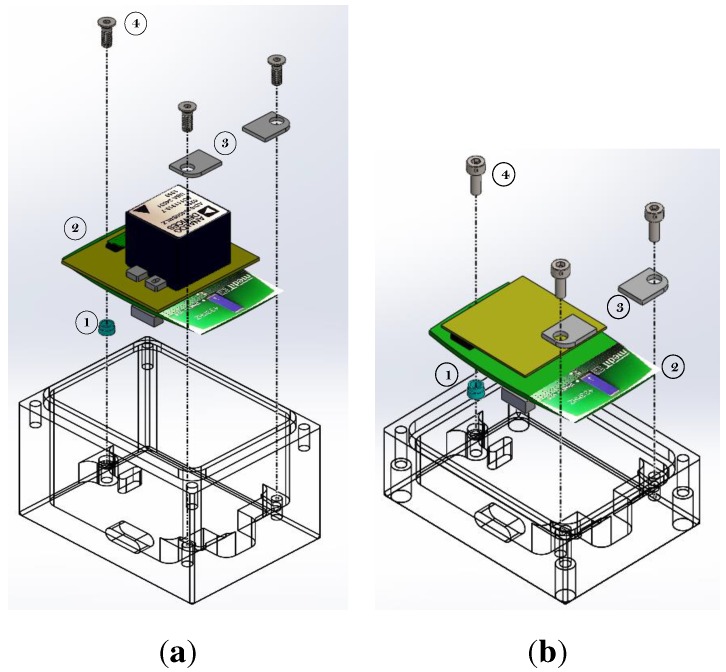
Design of the calibration case (1, Insert, aluminum; 2, 9DOF MARG sensor system; 3, Insert, plastic; 4, M3 screw, steel). (**a**) IPANEMA ADIS16400 arrangement; (**b**) IPANEMA EXO arrangement.

The case for the BSN nodes was fabricated from polymethyl methacrylate (PMMA) to prevent signal attenuation in the wireless communication of the BSN (or other wireless sensors that need to be calibrated). The case was designed to house two 9DOF MARG sensors at the same time. Both 9DOF MARG systems can be fixed by specifically-designed rectangular plastic plates and two mean screws in two corners of the top and lower part of the case. The opposite side of the board is fixed with a third screw and an aluminum cylinder. The arrangement for the fixation of the 9DOF MARG sensor systems in the calibration is shown in Figure 6 with the proper order to the assembly ADIS16400 and EXO board within the case.

### 3.4. Calibration Algorithm

For the sensor model given in Equation (13), it is now necessary to estimate the optimal parameters in order to calibrate the developed MARG sensor node properly. The calibration cube relates to an Earth-oriented navigation frame, which holds the gravity and the Earth’s magnetic field vector in the quaternion description as:

(16) $$\begin{array}{c} \begin{array}{cc} g_{q}^{n} & ={\left[\begin{array}{cccc} 0 & 0 & 0 & 1 \end{array}\right]}^{T}\;\\ h_{q}^{n} & =h\;{\left[\begin{array}{cccc} 0 & \cos(\alpha ) & 0 & \sin(\alpha )\; \end{array}\right]}^{T} \end{array} \end{array}$$

with amplitude *h* and inclination angle *α*. Capturing both EXO and ADIS data, in the first step, the cube is kept in resting position in order to determine the mean static bias vectors ${\tilde{\omega }}_{\text{stat}}$, ${\tilde{a}}_{\text{stat}}$, ${\tilde{m}}_{\text{stat}}\in R^{3}$ and ${\tilde{q}}_{\text{stat}}\in H_{1}$ for the different sensor types, where ${\tilde{q}}_{\text{stat}}$ results from the orientation estimated by the ADIS node. Taking into account the sensor’s dynamical characteristics, in the second calibration step, the cube is then moved by hand on an arbitrary trajectory and finally turned back into starting position, which yields the dynamic movement data of n∈N samples:

(17) $$\begin{array}{c} \begin{array}{cc} \tilde{\Omega } & =\left[\begin{array}{cccc} {\tilde{\omega }}_{\text{dyn},1} & {\tilde{\omega }}_{\text{dyn},2} & \ldots & {\tilde{\omega }}_{\text{dyn},n} \end{array}\right]\\ \tilde{A} & =\left[\begin{array}{cccc} {\tilde{a}}_{\text{dyn},1} & {\tilde{a}}_{\text{dyn},2} & \ldots & {\tilde{a}}_{\text{dyn},n} \end{array}\right]\\ \tilde{M} & =\left[\begin{array}{cccc} {\tilde{m}}_{\text{dyn},1} & {\tilde{m}}_{\text{dyn},2} & \ldots & {\tilde{m}}_{\text{dyn},n} \end{array}\right] \end{array} \end{array}$$

Therefore, the data obtained from the ADIS sensor node after orientation estimation with the EKF configuration derived in Section 3.2 provide both reference orientation $Q_{\text{ref}}$, as well as angular velocity $\Omega_{\text{ref}}$ with:

(18) $$\begin{array}{c} \begin{array}{cc} \Omega_{\text{ref}} & =\left[\begin{array}{cccc} \omega_{\text{ref},1} & \omega_{\text{ref},2} & \ldots & \omega_{\text{ref},n} \end{array}\right]\\ Q_{\text{ref}} & =\left[\begin{array}{cccc} q_{\text{ref},1} & q_{\text{ref},2} & \ldots & q_{\text{ref},n} \end{array}\right] \end{array} \end{array}$$

which are then used to determine the sensor model parameters. Regarding gyroscope characteristics, the first aim is to determine the offset from the static resting trials, yielding:

(19) $$b_{\omega }={\tilde{\omega }}_{\text{stat}}$$

Thus, the bias of the gyroscope data can be corrected by subtracting the static bias:

(20) $$\Omega =\left[\begin{array}{cccc} {\tilde{\omega }}_{\text{dyn},1}-b_{\omega } & {\tilde{\omega }}_{\text{dyn},2}-b_{\omega } & \ldots & {\tilde{\omega }}_{\text{dyn},n}-b_{\omega } \end{array}\right]$$

For further calculating the dynamical characteristics, in the next step, the matrix $T\in R^{3\times 3}$ needs to be found that satisfies the following minimization problem:

(21) $$\min_{T}{∥\Omega_{\text{ref}}-T\Omega ∥}_{2}$$

and can be obtained by:

(22) $$T=\Omega_{\text{ref}}\Omega^{+}$$

where $\Omega^{+}$ is denoted as the Moore–Penrose pseudo-inverse of Ω. Using polar decomposition [21], T then can be split giving:

(23) $$T=C^{b}\;K_{\omega }$$

**Figure 7:**
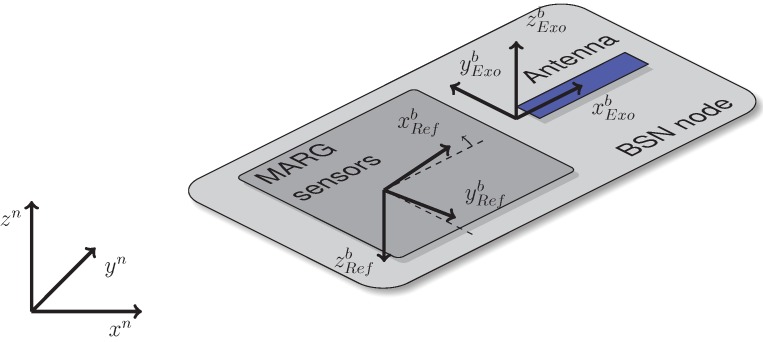
BSN node with mounted MARG sensor extension, different body coordinate frames $B_{Exo}$ and $B_{Ref}$ and calibration platform-based navigation frame *N*.

The DCM $C^{b}$ in Equation (23) describes the transformation from the ADIS reference body frame to the EXO body frame, which should approximately hold:

(24) $$C^{b}\approx \left[\begin{array}{ccc} \pm 1 & 0 & 0\\ 0 & \pm 1 & 0\\ 0 & 0 & \pm 1 \end{array}\right]$$

due to small mechanical or sensor package-based misalignments of the calibration cube, as depicted in Figure 7.

In the next step, the model parameters for both the accelerometer and magnetometer are obtained as follows. Using the previously-recorded quaternion-based orientation data $Q_{\text{ref}}$, the gravity and magnetic field vectors from Equation (16) are transformed via Equation (6) to the respective calibration cube orientations, yielding:

(25) $$\begin{array}{c} \begin{array}{cc} G_{q} & =Q_{\text{ref}}⊗g_{q}^{n}\;1_{1\times n}⊗{Q_{\text{ref}}}^{*}\\ H_{q} & =Q_{\text{ref}}⊗h_{q}^{n}\;1_{1\times n}⊗{Q_{\text{ref}}}^{*} \end{array} \end{array}$$

where $1_{1\times n}$ denotes a *n*-dimensional row vector of ones, and quaternion multiplication is performed column-wise. With Equation (23), the measured acceleration and magnetic field vectors are now rotated into the body reference frame:

(26) $$\begin{array}{c} \begin{array}{cc} A & =C^{b}\;\tilde{A}\\ M & =C^{b}\;\tilde{M} \end{array} \end{array}$$

Solving another convex optimization problem, the calibration parameters for both accelerometer and magnetometer can be obtained by the following optimization problems without constraints:

(27) $$\begin{array}{c} \begin{array}{cc} \min_{K_{a},b_{a}} & \;∥K_{a}A-b_{a}\;1_{1\times n}{-G∥}_{1}\\ \min_{K_{m},b_{m}} & \;∥K_{m}M-b_{m}\;1_{1\times n}{-H∥}_{1} \end{array} \end{array}$$

Note that both Equations (21) and (27) may be solved by using different optimization algorithms, like the interior-point, the trust-region-reflective or the Levenberg–Marquardt optimization. Within this contribution, the interior-point optimization was the algorithm of choice. At this point, we split our approach into two different procedures, which are evaluated in the next sections regarding their accuracy within different motion types. In the first approach, all columns of A that do not satisfy:

(28) $$1-\varepsilon <|{\tilde{a}}_{\text{dyn},i}|<1+\varepsilon$$

with a sufficiently high *ε* are neglected, as gravity is not measured correctly during accelerated movements. In the second approach, the acceleration samples were not neglected. This was done to further improve the calibration results for high dynamical movements, and thus, the reference gravitation was to be replaced by the reference acceleration $A_{\text{ref}}=\left[\begin{array}{cccc} a_{\text{ref},1} & a_{\text{ref},2} & \ldots & a_{\text{ref},n} \end{array}\right]$ measured by the ADIS node, such that:

(29) $$g_{i}=a_{\text{ref},i}$$

for every i∈{1,⋯,n} that does not fulfil Equation (28). In both cases, the true sensor outputs now can be obtained by solving Equation (13) for ***ω***, ***a*** and ***m***. As the first approach would provide the best results for slow movements, it was denoted as the static calibration procedure, where the second one, the dynamic calibration, is expected to deliver an orientation estimation within high dynamically experiments more precisely. Based on these assumptions, the derived calibration algorithms were compared to each other, as well as to an optical reference system, as shown in the following section.

## 4. Experimental Study and Results

**Figure 8:**
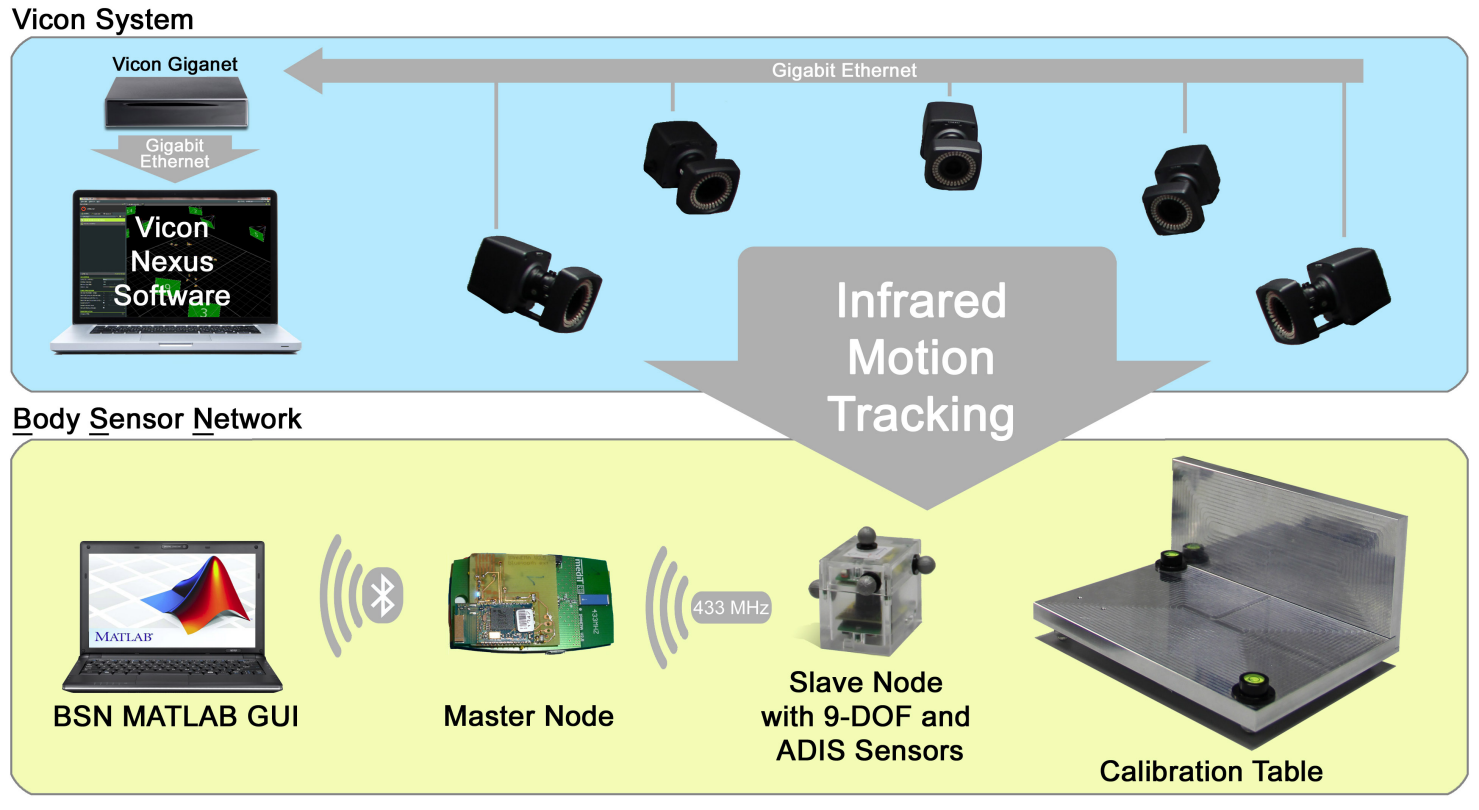
Overview of the components for sensor calibration.

The performance of the novel calibration setup and algorithm was validated in an experimental study. Here, an infrared motion tracking system (Vicon Bonita, 7 Cameras, Vicon, Oxford, UK) was used. Data from the BSN 9DOF MARG nodes was sampled at 75 Hz. The orientation of the reference case was tracked by using optical markers at a sampling rate of 100 Hz of the infrared motion tracking system. Figure 8 shows an overview of the experimental setup.

**Figure 9:**
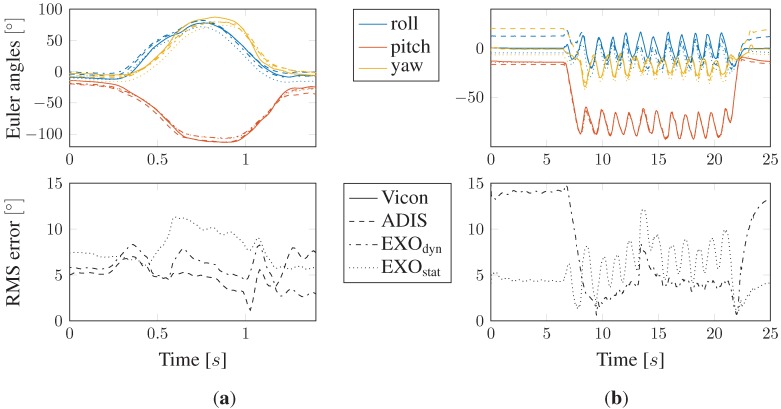
(**a**) Comparison of estimated orientation results *versus* real Vicon tracking data on arbitrary trajectory and RMS error of ADIS data, as well as dynamically, statically and non-calibrated EXO data and (**b**) the worst case scenario: comparison between the static and dynamic calibration algorithm within both fast and zero movements. The line styles are valid for all four graphs; the different colors specify the three possible orientations.

The optical markers for the motion tracking reference system were attached to the reference case, containing two 9DOF MARG sensor slave nodes. Before the start of the experiment and in the first step, the calibration table was aligned with the **g**-vector and magnetic north direction. In the second step, the inertial reference frame of the motion tracking reference system was aligned with the calibration table using an optical marker attached to the reference handle. In the first experiment, the ability of the ADIS reference node and the implemented orientation estimator should be validated to confirm the admissibility as an appropriate reference system for the calibration procedure. In resting position, the maximum orientation error was measured as ${\text{4.22}}^{{}^{\circ}}$, which was assumed as sufficient accuracy for applications with large positioning changes, like, for example, in body segment orientation tracking. Thus, the ADIS sensor node could be considered as an appropriate calibration reference system. In the next step, the performance of the proposed calibration algorithm was validated. Therefore, the calibration cube was repeatedly moved on an arbitrary trajectory inside the tracking space. Both the static and dynamic types of algorithms were applied on the obtained data. Figure 9a shows one time frame of the resulting estimated orientations against the real Vicon tracking data, both represented in the Euler angle description. Additionally, the root mean squared (RMS) error of all sensor nodes, respectively different calibration types, is depicted over time. Note that the RMS error in this case is meant to be a spatial performance criterion calculated as the root mean squared error over all three dimensions at each discrete sample time and, thus, varies over time. Obviously, the dynamic calibration algorithm in this case performs more precisely than the static one. Nevertheless, it is clearly shown that the proposed procedures are practically useful for calibration tasks, when comparing the results to the non-calibrated EXO orientation estimation error also depicted in Figure 9a. To further compare the performances of the static *versus* dynamic calibration procedure, in Figure 9b, the worst case scenario is presented. Here, the EXO board, on the one hand, was calibrated statically within slow calibration movements, whereas, on the other hand, the dynamical calibration was executed during highly frequent orientation changes. In applications with both zero movement phases and fast changes in orientation, the wrong calibration procedure would result in equally bad estimation performances. Furthermore, Figure 9b also gives an impression of the static calibrated estimation performance within motionless phases (0–6 s), respectively dynamically-calibrated orientation assessments within frequent changes (8–22 s).

In the last experiment, the performance of the proposed low cost orientation estimation system should be examined under realistic employment conditions. Therefore, the developed calibration cube was repeatedly moved on an arbitrary trajectory through the tracking space to imitate different types of motions. Calibration was performed both with the static and the dynamic calibration algorithm, such that the obtained results could be compared with each other. In Table 2, the statistical evaluation after the calibration of 10 consecutive experiments is shown. Note that these results are not reproducible in general in terms of temperature, number of experiments or mechanical deviations of the employed hardware, but may give an impression about the ability of the derived calibration procedure.

**Table 2:** Statistical evaluation of 10 consecutive calibration trials with arbitrary movements compared to the reference system.

	Dynamic Calibration			Static Calibration	
	RMS (rad)	(Mean, SD)		RMS (rad)	(Mean, SD)
roll	0.0614	−0.0350 ± 0.0456		0.0787	0.0177 ± 0.0639
pitch	0.0418	−0.0209 ± 0.0339		0.0473	−0.0063 ± 0.0456
yaw	0.0852	−0.0591 ± 0.0531		0.0733	0.0041 ± 0.0570
all	0.0653	−0.0383 ± 0.0246		0.0678	0.0051 ± 0.0363
Zhang and Yang:	**H + B Calibration**			**B Calibration**	
roll	0.0397	0.0014 ± 0.0397		0.0548	0.0252 ± 0.0487
pitch	0.0698	−0.0431 ± 0.0549		0.0854	−0.0171 ± 0.0836
yaw	0.0507	0.0060 ± 0.0503		0.0744	0.0095 ± 0.0738

## 5. Discussion

As the presented orientation estimation system will eventually be used to assess body segment orientations within biomechanical investigations, the proposed calibration algorithm showed promising results under realistic application conditions. The comparison between the low-cost MARG sensor and a high-precision optical tracking system yielded high correlation and maximum absolute RMS errors of ${\text{6.72}}^{{}^{\circ}}$. In practical experiments, the maximum RMS error after calibration in comparison to the reference system was ${\text{3.89}}^{{}^{\circ}}$ (Table 2). The obtained results have been reproducible within several experiments using equally-calibrated sensor nodes, which proves the reliability of this approach. As far as sensor nodes have not been calibrated using the dynamical approach with single highly dynamic motions during the calibration phase, the dynamical approach performs slightly better than the static one (Figure 9). This may be caused by dynamic side-effects that the acceleration sensor is exposed to within translational acceleration, which is not considered in the static implementation due to canceling out of all of the acceleration contributions that do not approximately equal one. The two proposed approaches were compared to each other in 10 consecutive calibration trials (Table 2). Though the dynamical approach delivered slightly better RMS results on average, we found the static calibration interestingly to be more accurate in offset cancellation of the remaining orientation error. This result could easily be obtained by comparing the mean value and standard deviation (SD) of the experiments. We assume that taking the reference acceleration data into account (Equation (29)), on the on hand, yields better results in terms of orientation estimation within highly dynamical movements, but on the other hand, also gives an additional offset, which could not be compensated by the implemented EKF. In addition to the discussion of the calibration procedure itself, the obtained results were compared to a similar work on this topic [4], where furthermore, approximately the same hardware configuration is used. As shown in Table 2, the results of the experimental study yielded comparable accuracy in orientation estimation in the three Euler angles. Nevertheless, it should be noted that both kinds of proposed algorithms (H + B calibration and B calibration only, where H is a transformation matrix comparable to K and B the combined bias like b) are not directly comparable to the proposed algorithms within this contribution. In addition, the protocol of the experimental setup, as well as the intended application purpose may have differed.

**Figure 10:**
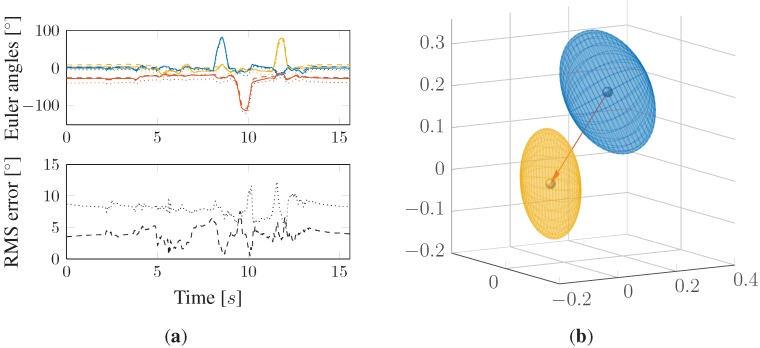
Exemplary calibration procedure measurement (${\text{90}}^{{}^{\circ}}$ rotation in each direction) and resulting errors compared to the ADIS reference system. (**a**) Non-calibrated (dotted) and calibrated (dashed) orientation estimation; (**b**) error distribution before (blue) and after (yellow) calibration.

The remaining error in absolute orientation estimation, as mentioned earlier, is one of the issues that needs to be discussed further. In Figure 10a, the resulting measurements of a calibration procedure are shown, where the calibration cube was rotated by ${\text{90}}^{{}^{\circ}}$ in each direction and then turned back in the starting position. As depicted in the lower diagram, after calibration, the remaining orientation error compared to the ADIS reference system is still about ${\text{4.5}}^{{}^{\circ}}$. An explanation for this is shown on the right side in Figure 10b, where the statistical parameters of the remaining orientation error are visualized in 3D space. Although the mean value, depicted by the balls in the middle, is shifted closer to the origin, the hull volume denoting the standard deviation nearly stays the same. This means that the error vector orientation varies approximately as before, and just the offset could have been reduced significantly. Thus, the use of time- and axis-related RMS in this case may be misleading in terms of evaluating the performance of error-minimizing calibration algorithms. Another interesting fact is that estimation errors are mainly caused by an offset in 3D space (orange arrow in Figure 10). Further investigations thus would be the minimization of non-offset-based errors and the source determination of these errors in terms of the hardware-based accuracy of each single sensor. If error sources are determined in our hardware solution, we will consider revising the sensor node, since more accurate sensor technology is already available. Until then, the main task would be to prove our system’s relevance in practical motion studies in comparison to standard optical tracking systems.

## 6. Conclusions

In this contribution, we presented a low-cost 9DOF MARG sensor node for our IPANEMA BSN. The obtained acceleration, angular velocity and magnetic field strength data were used to estimate the rigid body orientation via sensor fusion algorithms. To achieve better performance, we introduced a novel calibration algorithm, which takes into account orientation data from a body-fixed reference system. We presented a static and a dynamic calibration approach, both facilitated by our recently-developed calibration cube and platform. The measurement results were compared to a high-precision optical reference system. It was shown that our proposed algorithm significantly increases the accuracy of low-cost sensor devices and gives sufficiently precise orientation estimates. Thus, our developed system allows robust monitoring of body segment orientation during biomedical investigations, as also shown in an experiment. Future work will consider investigation of the error sources and separating hardware from algorithmic dependent errors in order to further improve the body segment tracking quality.
